# Progress in Surgery

**Published:** 1902-11-15

**Authors:** 


### Progress in Surgery.

*(Concluded from page 99.)*

### HERNIA.

**Obturator Hernia.**—Gladstone [8] reports an instance of double obturator hernia, each hernia of a rare form, observed in the dissecting room, and discusses the two methods of operative treatment, viz., the intra-abdominal and the extra-abdominal. He states that the two hernias in this case could have been dealt with easily by the intra-abdominal method through a single median laparotomy wound. It would be more difficult to expose obturator hernias by extra-abdominal incisions, owing to the depth and the proximity of the femoral vessels, but through such an incision the sac and its coverings, or any constriction by the cotylo-pubic band, could be more satisfactorily exposed and dealt with. The method would also be preferable if an artificial anus had to be formed.

**Strangulated Hernia.**—J. R. R. McCrindle [9] reports a case in which he successfully resected five inches of gangrenous bowel in operating for a femoral hernia which had been strangulated for 29 hours. A Murphy's button was used, and the resection performed through the groin incision after pulling down the intestine. The opening in the peritoneum was then sufficiently enla rged, the bowel reduced, and a radical cure performe d. At the time of operation the patient was in fair condition, and there was no abdominal distension, and the whole proceedings were completed in 35 minutes. On the following day a simple enema acted well, and on the sixteenth day the button was passed and the patient recovered. T. H. Kellock [10] reports a case of bilateral strangulated hernia in an infant. It is rare for strangulated inguinal hernia to be met with in infants under the age of one year. In the case in question a right inguinal hernia became irreducible for two days, and the child was fretful and vomited occasionally during this period. Herniotomy was performed. The knuckle of bowel found in the sac was very dark in colour, and deeply marked where it had been constricted, but there was no fluid in the sac. Two days later a hernia descended on the right side, and was reduced with difficulty. Nine days later this hernia descended again, and being irreducible was operated upon. The inguinal rings on both sides were very small, and had to be rather freely incised. No doubt the sacs were of funicular origin.

**Hernia of the Bladder.**—Willy Meyer [11] opened a discussion on this subject recently in the New York Academy of Medicine, and reported a case of the kind. The patient was a man of 60, with pronounced bronchitis an d an irreducible right inguinal hernia, so large that it prevented him walking. A radical operation was done under spinal anæsthesia. The analgesia was perfect. Despite all care a peculiar mass on the inner side of the sac was damaged, and a gush of urine confirmed the suspicion that this swelling was the bladder. The wound in the bladder was closed with two rows of sutures, and the neck of the sac partially closed with a continuous suture, leaving however an opening for the introduction of a gauze drain. A catheter was not left in the bladder. No complication ensued, and after 48 hours spinal anæsthesia was again induced, and the radical cure after the method of Bassini was completed. Recovery was uninterrupted. The patient was kept on his back six weeks. A truss was not recommnded. Six months later there was a slight recurrence of the hernia, caused by coughing, just where the vesical hernia had been pushed back. The patient now wears a truss, and is perfectly able to work as a carpenter. Statistics show that this complication of inguinal hernia occurs in about 1 per cent. of the cases. Spinal anæsthesia was only recommended when there was any reason for not employing ordinary anæsthetics. F. Kammerer stated that he had been surprised to note how well old and feeble persons stood spinal anæsthesia. C. L. Gibson said he had seen the bladder in hernia operations about ten times, though a number of these cases were not really examples of hernia of the bladder, but rather of a displacement of the viscus produced by pulling upon it during the operation.

**Hernia and Strangulation of the Vermiform Appendix.**—A. Barth [12] operated on a case of this kind recently. The patient, a woman 80 years of age, with no previous history of hernia, experienced a sudden pain in the right groin when lifting a weight. Symptoms of intestinal strangulation followed. On the 18th day, under anæsthesia, induced by the Schleich method on account of bronchitis and heart weakness, the swelling occupying the position of a femoral hernia was exposed by incision. On opening the sac stinking fluid escaped and a much swollen tightly strangulated vermiform appendix was found and removed. At the crural ring a small abscess and adhesions were present. The appendix was found to be perforated where it had been constricted, and to have a well-developed mesentery. The wound was sutured and healed well, but after some weeks the patient died. The case was obviously one of genuine strangulation of the appendix without primary inflammation. The symptoms in such cases must be referred to the pressure on the walls of the appendix, since there is no true intestinal obstruction established. Often the symptoms are mild. In the majority of cases of strangulation of the appendix the hernia is of the femoral variety.

**Hernia of Meckel's Diverticulum.** — R. E. Webster, of Ottawa,[13] reports an example of this affection. The patient was a female, aged 42. Three days before admission to hospital she had a fall, and had had severe abdominal pain and severe vomiting ever since. The abdomen was greatly distended, and a large tender mass occupied the left inguinal canal. The patient stated that she had frequently had a swelling there, which usually went back when she lay down. A portion of the hernia was easily reduced, but a hard mass still remained. The bowels moved shortly after, and the stools contained blood. The symptoms somewhat abated. At the operation the diverticulum, with a well-marked mesentery, was found and removed, and the opening left in the ileum, which admitted the tip of the finger, was closed with a double row of Lembert sutures. The diverticulum was $3\frac{1}{2}$ inches in length, the last half-inch being solid, and showing old inflammatory changes. A good recovery was made.

[8] Vide Abst. Brit. Med. Jour., Epitome 189, March 22, 1902. [9] Lancet, April 12th, 1902, p. 1,034. [10] Lancet, July 5th, 1902, p. 21. [11] Med. Rec., March 1, 1902, p. 353. [12] Vide Abst. Centralb. f. Chir., July 12, 1902, p. 765. [13] Annals of Surgery, April 1902, p. 503.

